# P53 - Smoking during pregnancy and asthma’s manifestation in childhood

**DOI:** 10.1186/2045-7022-4-S1-P108

**Published:** 2014-02-28

**Authors:** Paraskevi Maggina, Foteini Dareiotaki, Theodora Syriopoulou, Eleni Fourlani, Anastasios Koukouletsos, Ioanna Argyri, Aphroditi Kourti, Vaios Katsaros

**Affiliations:** 1 2nd Pediatric Unit, University of Athens, Athens, Greece; 2 Intensive Care Unit, General Hospital of Kalamata, Kalamata, Greece; 3 Pediatric Unit, General Hospital of Kalamata, Kalamata, Greece; 4 Pediatric Unit, Children's Hospital, Athens, Greece; 5 Scientific Associate, General Hospital of Kalamata, Kalamata, Greece

## Introduction

Mother's smoking during pregnancy has negative consequences in fetus respiratory system's development and asthma's manifestation later in children's life as well.

## Material

Recording the influence of mother's smoking during pregnancy to the frequency of asthma's manifestation in children.

## Methods

The study was performed at Pediatric Emergency Room and concerns the last two years (statistical analysis was done using the statistical package IBM SPSS Statistics v 19).

## Results

Presented in the following table.

**Table 1:** 

Smoking	Asthma		
	**No**	**Yes**	**Total**

**No**	210 (90.1%)	23( 9.9%)	233 (100%)

**Yes**	25 (67.6%)	12 (32.4%)	37 (100%)

**Total**	235 (87%)	35(13%0	270(100%)

## Conclusions

Smoking during pregnancy is asthma's predisposing factor at all ages. Mothers who smoked during pregnancy were three times more likely to acquire children suffering from asthma than those who did not smoke.

